# The Practice and Feasibility of Screening, Treatment, and Referral for Gaming Problems in Gambling, Alcohol and Other Drugs, and Youth Services

**DOI:** 10.1007/s11469-023-01010-4

**Published:** 2023-01-23

**Authors:** Jennifer J. Park, Daniel L. King, Laura Wilkinson-Meyers, Simone N. Rodda

**Affiliations:** 1 School of Population Health, University of Auckland, Private Bag 92019, 1142 Auckland, New Zealand; 2 College of Education, Psychology & Social Work, Flinders University, GPO Box 2100, 5001 Adelaide, South Australia Australia; 3 Department of Psychology and Neuroscience, Auckland University of Technology, 90 Akoranga Drive, Northcote, 0627 Auckland, New Zealand

**Keywords:** Gaming, Treatment, Prevention, Addiction, Screening, Brief intervention, Gambling

## Abstract

Screening, brief intervention, and referral to treatment (SBIRT) is a comprehensive approach to identifying people at risk of addiction, but its feasibility for gaming disorder is unknown. This study surveyed 88 clinicians from gambling, alcohol and other drugs, and youth services in New Zealand. Results indicated that the most frequent GD screening method was an unstructured interview (61%), but 74% stated they would use a standardized tool if available. Responsivity to the detection of GD was an immediate intervention (84%), and rates of referral were low (28%). Around 50% of clinicians indicated high confidence in administering motivational approaches and relapse prevention. There was strong support for screening training (85%), treatment guidelines (88%), self-help materials (92%), and access to internet-delivered CBT that could be used in conjunction with other treatment (84%). Clinicians appear motivated and willing to implement SBIRT for GD but report lacking necessary training and resources, including access to screening tools and treatment guidelines.

In 2019, gaming disorder (GD) was included as a diagnosis in the eleventh revision of the International Classification of Diseases (ICD-11) (World Health Organisation, 2019). GD is characterized by impaired control over gaming and continued use despite increasing negative consequences, including interference with psychological, social, occupational, and interpersonal life domains (Castro-Calvo et al., 2021). A recent meta-analysis reported that GD had a global prevalence of 3.05%, with higher rates for males compared to females (Stevens et al., 2021). GD frequently co-occurs with other addictions, including problem gambling and substance use disorders (Burleigh et al., 2019), as well as other mental health problems, including generalized anxiety disorder, depression, ADHD, social phobia, and obsessive-compulsive symptoms (Brooks & Clark, 2019; González-Bueso et al., 2018; Wang et al., 2017). Although the evidence base is still developing, the current recommendation for GD treatment is in-person cognitive behavioral therapy (CBT) (King et al., 2017; Stevens et al., 2019; Wölfling et al., 2019). Many people with GD will not have access to in-person treatment due to cost or accessibility barriers to specialist services, particularly in the COVID-19 context (King et al., 2022), which has prompted calls for a broader range of options, including brief interventions (Park et al., 2021).

Screening, brief intervention, and referral to treatment (SBIRT) is a widely used approach to early intervention and the treatment of substance use disorders (Babor et al., 2007). SBIRT is consistent with a public health approach to reduce gaming harm because they provide universal screening and immediate responses (Rumpf et al., 2018). SBIRT aims to identify people at risk for substance use disorders and those who have already developed a problem. This approach uses opportunistic and rapid screening in settings and situations when the person is not actively seeking assistance for their problem. When screening detects moderate risk for problems, an immediate brief intervention or brief treatment is provided (Agerwala & McCance-Katz, 2012; Room et al., 2005). Where severe risk or potential dependency is identified, there is a referral to specialist treatment. Reviews indicate that SBIRT may decrease healthcare costs and maximize resource utilization when delivered in a range of mental health services, addiction services, and medical settings such as primary care, emergency departments, and schools (Agerwala & McCance-Katz, 2012; Aldridge et al., 2017; Broyles & Gordon, 2010). Several studies have indicated reductions in alcohol and illicit drug use 6 months after the intervention (Agerwala & McCance-Katz, 2012; Del Boca et al., 2017). SBIRT may also be effective for alcohol reduction in adolescents, with a meta-analysis indicating that modest beneficial effects may persist for up to 1 year following the intervention (Tanner-Smith & Lipsey, 2015).

SBIRT may be feasible for gaming disorder if there are valid and reliable screening tools and if these tools are routinely administered. Reviews of screening and assessment tools for gaming disorder indicate that tools are generally free and accessible and have been developed based on general population surveys and convenience samples (King et al., 2020b; Stevens et al., 2021). These measures assess the ICD-11 criteria with a focus on functional impairment (e.g., employment or relationships) and prioritization of gaming, unsuccessful attempts to change, and gaming to escape negative mood (King et al., 2020a, b). Even though valid and reliable screening tools are available, studies suggest a range of barriers can prevent implementation. Barriers include lack of time and/or resources, competing priorities, patient responsiveness, and lack of training or perceived competency to deliver screening and brief interventions (Johnson et al., 2011; Rodda et al., 2018a; Rosário et al., 2021). One study has examined barriers to screening specifically for gaming disorder. Dullur and Hay (2017) surveyed 142 psychiatrists in Australia and New Zealand and found that about 60% reported never or rarely screening for GD. Reported barriers included the lack of (i) acceptance of the concept of GD, (ii) time, and (iii) confidence to conduct screening.

Multiple reviews indicate that treatment is effective for GD (King et al., 2017; Stevens et al., 2019; Zajac et al., 2017), but there is much less evidence for brief interventions (Park et al., 2022). A review of GD treatment by Stevens et al. (2019) indicated that CBT was effective in the short term in reducing GD symptoms as well as co-occurring depression and anxiety. Across the 13 included studies, treatment was delivered in individual and group settings and consisted of 8 or more sessions delivered by a psychologist or psychiatrist. Park et al. (2022) examined the effectiveness of internet-delivered treatment for behavioral addictions. Four studies of specific brief interventions for gaming disorder were identified, including three RCTs and one pre-post study. Preliminary effectiveness was mixed, with two studies indicating short-term improvements to duration or severity and one study indicating similar symptom improvements compared to in-person treatment. Park et al. (2022) also examined the content of interventions and reported that gaming studies focused on cognitive restructuring (*n *= 3), exposure therapy (*n *= 1), and a combination of motivational and relapse prevention techniques with social support and feedback on assessment (*n* = 1).

## The Present Study

Early identification plays an important role in preventing the emergence of gaming problems. SBIRT may be a useful approach to detect and respond to gaming disorder when administered in addiction or youth services where people are likely to present for co-occurring conditions. New Zealand has publicly funded gambling, alcohol and other drugs (AOD), and mental health services that offer free or low-cost brief interventions, treatment, and referral, delivered in-person, online, and via phone (Patterson et al., 2018). Services are widely available for adults and adolescents in community settings and are provided by psychologists, social workers, and counsellors. Professional development for addictions screening and treatment is provided through organizations such as Te Pou and the Addiction Practitioners Association Aotearoa New Zealand (DAPAANZ), which offer continuing professional development through webinars, online training modules, and in-person events such as an annual conference. DAPAANZ also offers accreditation to practice addictions work in New Zealand which involves a combination of supervised practice and a professional addiction-related academic qualification. To our knowledge, no services specialize in gaming treatment, nor does professional accreditation include a focus on gaming screening or treatment. Anecdotally, addiction services report gaming-related presentations and the co-occurrence of GD with gambling, alcohol, and other drug problems.

The current study sought to determine the current practice and feasibility of SBIRT in New Zealand. The study aimed to determine (i) the barriers and facilitators to screening GD and the frequency and method of screening administration, (ii) responsivity to detecting GD, (iii) confidence to administer change techniques to address GD, and (iv) experience and preferences for training and resources.

## Methods

### Participants and Recruitment

Participants were recruited from a range of publicly funded gambling, AOD, and youth services across New Zealand. Included service providers were selected on the basis that they provide addiction screening, brief intervention, or treatment to adults and adolescents. An invitation to participate was emailed in June 2021 to the managers and team leaders of 90 providers, inclusive of gambling (*n *= 16), AOD (*n *= 44), and youth services (*n *= 30). Managers and team leaders were asked to forward the invitation to clinical staff. An inclusion criterion stipulated that clinicians needed to have at least one client interaction about internet-enabled addictions, inclusive of gaming, pornography use, internet gambling, or social media use. The current study was part of a larger survey of internet-enabled addictions, and this inclusion criterion was set so that the study sample was likely to have some knowledge of potential screening, brief intervention, and referral practice. Eligible participants completed a 20-minute survey and were reimbursed with a $40 shopping voucher and a free copy of the research team’s client guidelines for gaming reduction. The study was approved by the University of Auckland Human Participants Ethics Committee (Reference: UAHPEC3449).

A total of 88 clinicians completed the survey, drawn from 35 different gambling, AOD, and youth services. As indicated in Table 1, clinicians were most frequently female, with an average age of 44 years. The sample reported extensive experience working with people with addiction (over ten years on average). The most frequent professions were AOD workers (*n* = 24, 27.3%), counsellors or psychologists (*n *= 22, 25.0%), and social workers, case workers, or occupational therapists (*n *= 20, 22.7%). Participants reported that their service was funded to provide screening and treatment for gambling (*n *= 61, 69.3%), AOD (*n *= 60, 68.2%), tobacco reduction (*n *= 34, 38.6%), and gaming (*n *= 22, 25.0%). Client groups were typically adults (*n* = 80, 90.9%) and adolescents (*n* = 53, 60.2%), with 20 (22.7%) clinicians reporting their client group included children. Clinicians reported they were funded to tailor their service or practice for specific cultural groups including Māori (*n *= 75, 85.2%), Pacific (*n *= 71, 80.7%), and Asian people (*n *= 66, 75.0%).

**Table 1:** Participant sociodemographic characteristics, profession, and expertise (*N* = 88)

Variable	Data (*n*, %)
Age (*M, SD*)	44.4 (12.3)
Gender - Female	55 (62.5)
Profession	
AOD worker	24 (27.3)
Counsellor/Psychologist	22 (25.0)
Social worker/Case worker/Occupational therapist	20 (22.7)
Manager	8 (9.1)
Nursing/Medical	6 (6.8)
Other	8 (9.1)
Years working with addiction (*M, SD*)	10.5 (8.3)
Years of experience in current role (*M, SD*)	7.4 (6.4)
Employment status	
Full-time	75 (85.2)
Part-time/Casual	13 (14.7)
Education	
Postgraduate degree	41 (46.6)
Undergraduate degree	34 (38.6)
Certificate/Diploma/Secondary education	13 (14.8)
Ethnicity	
New Zealand European	42 (47.7)
Pacific	20 (22.7)
Māori	14 (15.9)
Asian	10 (22.7)
Middle Eastern/Latin American/African	2 (2.3)

### Measures

The survey collected information on demographic characteristics, level of education, and experience in addictions treatment. Participants were asked to estimate the frequency of gaming presentations on a 5-point scale (*never* to *very often*) and the approximate number of clients each month. Previous training in screening, treatment, and referral for gaming problems was examined over the past five years. Participants were also asked to indicate their interest in (i) screening training, (ii) treatment guidelines, (iii) self-help materials, and (iv) internet-delivered CBT that can be used with a client for treating GD.

Barriers and facilitators were assessed with a 16-item tool that examined attitudes to screening in mental health and addiction services (Manning et al., 2020). The tool included barriers, such as the attitudes towards GD as a clinical disorder and the perceived importance of treating GD. It also included facilitators, such as funding for screening and the importance and availability of a suitable screen. Each response was measured on a 5-point scale (*strongly disagree* to *strongly agree*). Participants also rated their comfort with screening on a 4-point scale from *very uncomfortable* to *very comfortable*.

Responsivity to GD was measured with a 7-item tool that assessed actions taken, should GD be detected. On a 5-point scale (*never* to *always*), participants indicated whether they would (i) conduct further assessment, (ii) refer to pharmacotherapy, (iii) provide psychological treatment, (iv) address immediate harm, (v) refer to another agency, (vi) offer self-help materials, or (vii) take no further action. Responsivity was also assessed through an examination of the types of behavior change techniques (BCTs) that clinicians would deliver for GD and their level of confidence in administering each technique (4-point scale of *would not use* to *very confident*). Nineteen BCTs relevant to GD were adapted from a categorization tool developed for problem gambling (Rodda et al., 2018b). As indicated in Table 2, participants were provided brief summaries of each BCT based on a previous review of BCTs for internet-enabled addictions (Park et al., 2022). For ease of comprehension, BCTs were organized into the categories of assessment and psychoeducation (2 items), motivational enhancement (5 items), cognitive and behavioral (8 items), and skill-building (4 items). 

**Table 2:** Gaming-related BCT items and descriptors

Theme	Technique	Description as applied to gaming treatment
Assessment and psychoeducation	Information gathering	An assessment or strategic questions to understand the nature of the problem
	Information provision	Information provided about problem gaming, harm, risk factors, and psychology of addiction
Motivational	Decisional balance	Consider pros and cons of behavior change and imagine positive outcomes of change
	Goal setting	Set a goal to limit, reduce, or quit gaming or decide on the types, duration, and frequency of gaming
	Feedback on assessment	Summary of data collected against a standard such as a cut off score of problem gaming
	Motivational enhancement	Increase change talk and problem awareness. Strengthen commitment and self-efficacy
	Social comparison	Planned comparison of gaming behaviors, such as frequency and time spent, with other social groups
Cognitive-behavioral	Behavioral substitution	Substitution of gaming for non-problematic behaviors
	Cognitive restructuring	Erroneous thoughts and beliefs are challenged, and more adaptive alternatives are generated
	Exposure	Systematic, gradual, and controlled exposure to gaming situations and cues with the purpose of extinguishing urges
	Imaginal desensitization	Progressive application of relaxation when intentionally exposed to a gaming-related stimuli, image, or visualization
	Problem-solving	Identification, generation, and implementation of solutions
	Relapse prevention	Identification and prevention of high-risk situations, triggers, and engagement in unplanned gaming
	Self-monitoring	Prompt a record, diary, or other means of recording thoughts or behaviors over a specific period
	Stimulus control	Modify the environment to reduce access to gaming and avoid social cues
Skills development	Financial management	Budgeting, or banking systems and financial management
	Social skills training	Assertiveness, communication, and interpersonal skills
	Mindfulness	Focusing attention in a non-judgemental way on moment-by-moment thoughts, feelings and actions
	Social support	Seek practical and emotional support from others

### Data Analysis

Descriptive statistics (i.e., means, standard deviations, frequency, percentage) were calculated for participant demographics, consultations and caseload, screening, attitudes towards GD screening, responsivity to GD detection, and training and resource needs. When clinicians estimated their caseload in ranges (e.g., 20–25), the midpoint value was applied for analysis. To aid the interpretation of findings, response options for barriers and facilitators were collapsed into smaller categories (*strongly disagree/disagree*, *neutral*, and *strongly agree/agree*). Quantitative analysis was conducted with SPSS software (version 27).

## Results

### Frequency of Presentations for Gaming Problems

**Fig. 1:**
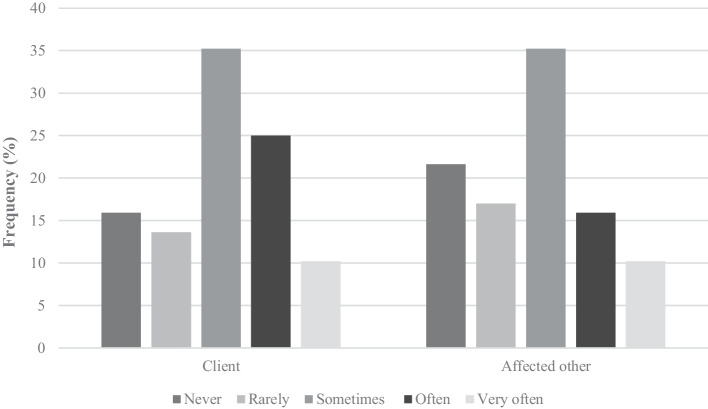
Frequency of consultations with a client or affected other about gaming problems

Almost all clinicians had at least one consultation in the past year with a person experiencing gaming problems (*n* = 74, 84.1%) or a person affected by someone else’s gaming (*n* = 69, 78.4%). Figure 1 shows that around 35% of clinicians had often or very often had consultations about gaming. Clinicians also reported that the average monthly caseload for gaming-related problems was 4.1 (SD = 7.7), which was a mix of adults and adolescents. Figure 1 also shows that 26% of clinicians had consulted with the family or friends of someone with a gaming problem. Clinicians reported an average monthly caseload of 2.7 (SD = 4.0) for affected others impacted or concerned about another person’s gaming.

### Barriers, Facilitators, and Approach to Screening GD

Participants were asked about their general level of comfort with screening for GD. Sixty-five clinicians (73.9%) indicated they were somewhat or very comfortable with screening, with 26.1% being somewhat or very uncomfortable. Clinicians reported that their primary course of action to screen for GD was to conduct an unstructured interview (*n *= 54, 61.4%), with some using a standardized tool (*n *= 14, 15.9%). Nine (10.2%) participants indicated that their service administered screening for GD as part of the intake assessment. Other approaches were to combine an unstructured interview with an administration of a screening tool (*n *= 4) or screen for other issues such as problems with family, sleep, social life, and obsessive thoughts about gaming (*n *= 7). There was broad agreement that GD was a clinical disorder (76.1%) and that it co-occurred with other issues, which rendered screening important (70%). Almost all agreed or strongly agreed that there should be funding for services to screen for GD (75%).

As indicated in Table 3, the most frequently endorsed barrier was not being aware of available screening tools as well as the perception that people accessing gambling, AOD, and youth services would not want to be screened for GD. Around one in five agreed or strongly agreed that GD detection did not require a formal screen and/or that screening and referral for GD was not part of their role. Participants agreed or strongly agreed that if they had access to a recommended screening tool, they would use it and that a brief screen would be a useful part of routine clinical practice. Almost three-quarters agreed that it was important to identify GD. 

**Table 3:** Barriers and facilitators for GD screening (*N* = 88)

Item	Strongly disagree/disagree	Neutral	Strongly agree/agree
GD is not really a clinical disorder	67 (76.1)	14 (15.9)	7 (8.0)
Governments should fund my service to screen for gaming disorder	1 (1.1)	21 (23.9)	66 (75.0)
If I had access to a recommended screening tool for GD I would use it	3 (3.4)	20 (22.7)	65 (73.9)
A brief GD screen would be a useful part of my routine clinical practice	3 (3.4)	22 (25.0)	63 (71.6)
It is important to identify GD among my clients	3 (3.4)	22 (25.0)	63 (71.6)
There is no point conducting screening as my service does not treat GD	63 (71.6)	12 (13.6)	13 (14.8)
GD does not co-occur with other issues often enough to bother screening	62 (70.5)	18 (20.5)	8 (9.1)
There are too many more important issues to screen for than GD	60 (68.2)	21 (23.9)	7 (8.0)
To treat GD, there needs to be agreement between the client and me that gaming is an issue	9 (10.2)	21 (23.9)	58 (65.9)
There is not enough time to conduct screening for GD in my workplace	52 (59.1)	26 (29.5)	10 (11.4)
My sector should introduce screening for the whole service system	4 (4.5)	36 (40.9)	48 (54.5)
Use of standardized screening tools is only necessary if a client mentions gaming	47 (53.4)	29 (33.0)	12 (13.6)
Screening and referral for GD is not part of my job	46 (52.3)	26 (29.5)	16 (18.2)
Detecting GD does not require a formal screen	37 (42.0)	34 (38.6)	17 (19.3)
People accessing treatment do not want to be screened for GD	36 (40.9)	32 (36.4)	20 (22.7)
I am aware of what screening tools are available to me for detection of GD	40 (45.5)	25 (28.4)	23 (26.1)

### Responsivity to the Detection of GD

Participants reported their responsivity to the detection of GD. Most clinicians reported that if a client disclosed a gaming problem, they would screen or offer treatment or referral (*n* = 77, 87.5%). Clinicians would also often or always offer to address the immediate gaming-related harm (*n *= 74, 84.1%) and/or conduct further assessment (*n* = 71, 80.7%). Seventy-one percent stated that they would often or always offer self-help materials, and almost half of the respondents would often or always provide psychological treatment (*n *= 41, 46.6%). Just one-quarter would often or always refer to another agency (*n *= 25, 28.4%) and most would never or rarely refer to pharmacotherapy (*n *= 62, 70.5%).

Nineteen BCTs were assessed for their use in responding to or treating GD. Almost all BCTs that had been used for gambling treatment were deemed suitable for GD. As indicated in Table 4, BCTs that would not be used by some clinicians included financial management (*n *= 9, 10.2%), social comparison (*n *= 9, 10.2%), exposure therapy (*n *= 6, 6.8%), and imaginal desensitization (*n *= 5, 5.7%). Clinicians rated themselves as very confident in gathering information and, to a lesser extent, information provision. Clinicians also rated themselves as very confident in delivering motivational approaches, relapse prevention, and problem-solving as well as offering skill-building approaches like mindfulness and social skills training. There were lower levels of confidence in techniques that relied on gaming-specific knowledge, including exposure therapy, imaginal desensitization, feedback on assessment, and social comparison.

**Table 4:** BCT usage and confidence in responding to GD (*N* = 88)

Change techniques	Would not use	Not confident	Moderately confident	Very confident
Assessment and psychoeducation				
Information gathering	1 (1.1)	2 (2.3)	43 (48.9)	42 (47.7)
Information provision	1 (1.1)	7 (8.0)	45 (51.1)	35 (39.8)
Motivational approaches				
Motivational enhancement	2 (2.3)	4 (4.5)	34 (38.6)	48 (54.5)
Goal setting	1 (1.1)	4 (4.5)	35 (39.8)	48 (54.5)
Decisional balance	2 (2.3)	0 (0.0)	49 (55.7)	37 (42.0)
Feedback on assessment	3 (3.4)	18 (20.5)	43 (48.9)	24 (27.3)
Social comparison	9 (10.2)	10 (11.4)	45 (51.1)	24 (27.3)
Cognitive and behavioral approaches				
Relapse prevention	1 (1.1)	2 (2.3)	45 (51.1)	40 (45.5)
Problem-solving	1 (1.1)	4 (4.5)	45 (51.1)	38 (43.2)
Self-monitoring	2 (2.3)	2 (2.3)	51 (58.0)	33 (37.5)
Stimulus control	3 (3.4)	9 (10.2)	44 (50.0)	32 (36.4)
Behavioral substitution	1 (1.1)	4 (4.5)	53 (60.2)	30 (34.1)
Cognitive restructuring	2 (2.3)	6 (6.8)	50 (56.8)	30 (34.1)
Exposure	6 (6.8)	29 (33.0)	43 (48.9)	10 (11.4)
Imaginal desensitzation	5 (5.7)	27 (30.7)	48 (54.5)	8 (9.1)
Skill-building approaches				
Mindfulness	1 (1.1)	4 (4.5)	43 (48.9)	40 (45.5)
Social skills training	1 (1.1)	6 (6.8)	42 (47.7)	39 (44.3)
Financial management	9 (10.2)	14 (15.9)	42 (47.7)	23 (26.1)
Plan social support	1 (1.1)	2 (2.3)	50 (56.8)	35 (39.8)

Twenty-four participants listed additional treatment approaches that were not offered in the list of BCTs. The most common approach was the exploration of identity, values, beliefs, well-being, and spirituality (*n *= 8, 33.3%), followed by a family-focused approach (*n *= 7, 29.2%), Te Whare Tapa Whā (health model for understanding Māori health) (*n *= 4, 16.7%), and acceptance and commitment therapy (*n* = 2, 8.3%).

Participants were also asked to report on relevant BCTs for working with child and adolescent populations. Across the sample, 39 clinicians endorsed items from the BCT list or added their own techniques. The most frequently endorsed BCTs were planning social support and family involvement (*n* = 17, 43.6%), motivational enhancement (*n *= 8, 20.5%), cognitive restructuring (*n *= 6, 15.4%), behavioral substitution (*n *= 6, 15.4%), and relapse prevention (*n *= 2, 5.1%). Other approaches for children and adolescents were drawing, art, or other creative therapy (*n *= 8, 20.5%), addressing underlying issues (*n *= 4, 10.3%), and Te Whare Tapa Whā (*n *= 2, 5.1%).

### Training in Screening and Resource Needs for Gaming Problems

Participants reported on previous training and professional development for gaming screening, treatment, and referral. A total of 58% had attended a general information session, but most had not been provided training for GD screening. Rates of training for GD screening (33%) were similar to training for the provision of GD treatment (38%). There was strong support for training in screening (*n* = 75, 85.2%), treatment guidelines (*n *= 77, 87.5%), self-help materials (*n *= 81, 92.0%), and internet-delivered CBT that can be used with a client for treating GD (*n *= 74, 84.1%).

## Discussion

This preliminary study is the first to explore the practice and feasibility of SBIRT for GD. In a sample of clinicians providing screening, treatment, and referral for gaming-related issues, the current study found GD presentations were common in gambling, AOD, and youth services in New Zealand. Of the clinicians with prior experience with internet-enabled behavioral addictions, 84% reported consulting clients presenting with gaming problems and a further 78% reported consulting affected others. Notably, these figures were based primarily on participants’ clinical judgement because most (61%) clinicians reported that detection was via an unstructured interview rather than a psychometrically valid tool.

Clinicians were largely supportive of screening for GD but reported that there were barriers to address. Clinicians strongly agreed that GD was a clinical disorder and that routine screening would be helpful for clinical practice. Most of the sample indicated that screening could be supported through government funding like reimbursements for other mental health and addiction screening. The most frequent reported barrier was a belief that a client may not want to be screened. This finding is consistent with a previous study in problem gambling (Rodda et al., 2018a) which reported a perception of Australian mental health service providers that screening for other co-occurring issues might affect therapeutic alliance (e.g., embarrass the client). Unfortunately, this approach can mean that the person is not screened for GD, especially where gaming behaviors are not reported.

Regarding responsivity, most clinicians in addiction and mental health services would apply an immediate intervention if GD were detected. The present study administered a set of BCTs that were previously identified as techniques used to treat problem gambling (Rodda et al., 2018b) and found that almost all clinicians rated these techniques as relevant to responding to GD. BCTs are often used for behaviors that share similar psychological mechanisms of action to gaming. For gambling, alcohol consumption, and binge eating, the most frequently administered BCTs have been identified as motivational (e.g., feedback on behavior), cognitive and behavioral (e.g., problem-solving), and social (e.g., social comparison) (Humphreys et al., 2021). Exposure therapy and imaginal desensitization were rated as the least appropriate for gaming disorder which may reflect the absence of evidence on the effectiveness of these techniques for GD.

There were at least moderate levels of confidence in delivering a range of different BCTs that spanned motivational to cognitive and behavioral techniques despite very limited training or professional development. In the current study, clinician confidence was weakest for techniques like information provision, feedback on assessment, and social comparison that required gaming-specific knowledge. The absence of training is a problem because clinicians may miss or underestimate important clinical information that is specific to gaming and be unable to deliver treatment that relies on gaming-specific knowledge (e.g., CBT). This is also important from a consumer perspective whereby a previous study involving treatment seekers reported concerns that providers did not fully understand gaming mechanisms, culture, or social aspects. In some cases, consumers felt stigmatized or deterred from seeking help (Park et al., 2021). Professional development should therefore include extensive education around gaming, including gaming culture, social aspects, and how gaming works.

The present study found that just over one-quarter of the sample referred clients with gaming problems to another treatment or support option. This finding likely reflects a lack of clear referral pathways for gaming in New Zealand, where no specialist or government-funded gaming treatment services exist. Ideally, people with gaming problems have access to a comprehensive healthcare approach that includes free information, support, and treatment that is easily accessible and tailored to individual needs (Park et al., 2021). The emergence of a comprehensive approach should be co-designed, developed, and evaluated in conjunction with consumers to ensure that it is relevant, appropriate, and effective. It should consider a whole system approach inclusive of (i) phone, online, and in-person options, (ii) automated screening tools for gaming and comorbidities, and (iii) a wide range of brief intervention and treatment types (Rodda et al., 2022). Ideally, the system should have tailored support for people across the spectrum of problem severity. Tailored support for low and moderate severity may be in the form of self-help resources and support for how people naturally recover, such as self-monitoring, urge management, and maintaining readiness (Rodda et al., 2018c). Support should extend to parents and other family and friends who want to help someone with gaming problems or who are impacted by another person’s gaming. Support might include methods to restructure environments and stimulus control, persuasions, and monitoring (Gong & Rodda, 2020). Affected others is an overlooked field of study with a recent review indicating that there have been no RCTs investigating affected other treatment for gaming disorder (Merkouris et al., 2022).

### Is SBIRT Feasible for Gaming Disorder?

SBIRT may be an appropriate model for gaming disorder, but the current study indicates much work is needed before the model is feasible. A key issue is the lack of gold-standard screening and diagnostic instruments for gaming disorder (King et al., 2020b; Long et al., 2021). There is an even larger gap for rapid and brief screening or screening tools that are appropriate for administration in clinical services. Future research should develop an efficient and easy-to-administer GD screening tool that can be readily adopted by clinicians. Although there is much work in the development and testing of interventions for gaming (King et al., 2017; Stevens et al., 2019), there is much less evidence for brief interventions (Park et al., 2022). In other areas of addiction studies, motivational approaches are frequently delivered as brief interventions which have been shown to improve symptoms and increase treatment readiness (DiClemente et al., 2017). Given clinician confidence in delivering motivational approaches, this may be a promising area of inquiry. Clinicians reported that they would be interested in internet-delivered CBT that could be delivered in conjunction with other treatment. Research on barriers and facilitators to SBIRT in alcohol studies identified the use of technology as an important method of addressing the complex array of presenting issues (Abidi et al., 2016). The benefit of a blended treatment approach is that clinicians can continue with treatment as usual for the presenting issue and simultaneously support the client to access internet-delivered CBT for GD.

A major challenge for SBIRT for gaming is that screening, intervention, and referral pathways are currently weak. For example, we found a low referral rate to other treatments, which may reflect multiple compounding issues, including inadequate screening, confidence in delivering a brief treatment, and unclear referral pathways. Due to the low rate of standardized screening, it is possible that the severity of gaming is not detected and resolving gaming problems is therefore not a clinical priority. It is also possible that in the absence of professional development or in-depth knowledge of gaming, clinicians believe they can successfully administer techniques that are effective for other addictions, and therefore, referral is not warranted. The low referral rate also raises questions as to whether there is routine screening for depression, anxiety, and other addictions which co-occur with gaming problems (Burleigh et al., 2019) and how and when the person receives treatment. To support SBIRT, guidelines that include screening, brief intervention, and referral need to be developed and their effectiveness evaluated.

The current study involved clinicians working in addiction services. However, SBIRT in alcohol studies has focused on screening across a wide range of settings, inclusive of allied health, to broaden reach and capacity (Broyles & Gordon, 2010). SBIRT for alcohol involves delivery by a wide range of professionals, such as general practitioners, nurses, and those in settings such as emergency care. It may be that these settings are not appropriate for GD screening, but other settings should be identified, such as schools and higher education (Rumpf et al., 2018; Yen et al., 2022). Research in SBIRT for alcohol reduction suggests training and resources to support implementation may need to be tailored according to professional skills and competencies, culture, and individual preferences (Wamsley et al., 2018).

If SBIRT is feasible, further work is also needed to evaluate and guide implementation. Behavior change is difficult, and our study indicates that clinicians have already adopted their own unique approaches to screening and treatment that may be resistant to change. The Theoretical Domains Framework (TDF) is a widely used approach to identifying implementation challenges (Atkins et al., 2017). The TDF presents 14 domains, including health professional knowledge and skills, beliefs and attitudes, resources, motivation, and behavioral regulation. The framework has been used to guide the implementation of other SBIRT for addiction. For example, one review on screening for harmful alcohol use in clinical settings reported clinician bias related to alcohol health literacy, knowledge of consumption guidelines, the degree of comfort in screening, and the perception of client willingness to be screened (Johnston, 2022). Another study on SBIRT for smoking cessation also reported deficits in knowledge inclusive of counselling methods and referrals, beliefs about capabilities and confidence to screen, and environmental context and resources like lack of time, training, and material information (Merianos et al., 2022). Attempts to implement SBIRT for gaming should consider each of these issues identified in the TDF prior to implementation and evaluate the degree to which they have been addressed.

### Study Limitations

This is the first study to take an in-depth look at screening, treatment, and referral, but caution is recommended due to a range of limitations. First, the study could not estimate the true rate of gaming-related presentations in addiction services because it only included clinicians who had provided care to at least one person impacted by internet-enabled addictions. One way to address this issue is with a clinical audit, but this approach is unlikely to be valid or reliable until routine screening for GD is introduced. Second, the sample was self-selected and drawn from a range of addiction and youth services in New Zealand. Self-selection means that the experiences reported in the current study may not be representative of the wider workforce in New Zealand or internationally. The study invited clinicians from 90 different services and received responses from 35 services, but it is likely that many non-responders did not meet the inclusion criteria rather than did not respond to the invitation. Third, we identified that clinicians use a range of different tools when screening for GD. However, we did not ask about the types of tools used. Future research should determine what exact tools clinicians use to screen GD and how they found out about it. Fourth, we did not ask about the duration of treatment episodes when working with people with gaming problems. Thus, it is unclear whether the person receives a brief intervention or treatment and the nature of that treatment. Future research should consider qualitative approaches with clinicians and consumers to gain a better understanding of patient pathways and how and when SBIRT is most appropriate and effective.

## Conclusion

SBIRT is already a widely used approach for substance use disorders (Babor et al., 2007) and has the capacity to make a difference to people affected by gaming problems by expanding screening activity and options for treatment. The New Zealand addictions workforce indicates readiness and willingness to implement SBIRT but is hampered by multiple resource and professional development limitations. To support the implementation of SBIRT, clinicians require a valid, brief, and rapid screening tool, and evidence-based guidelines and resources to provide an immediate brief intervention.
